# Functional characterisation of a new halotolerant seawater active glycoside hydrolase family 6 cellobiohydrolase from a salt marsh

**DOI:** 10.1038/s41598-024-53886-4

**Published:** 2024-02-08

**Authors:** Daniel R. Leadbeater, Neil C. Bruce

**Affiliations:** Centre for Novel Agricultural Products, Department of Biology, University of York, Heslington, York, YO10 5DD UK

**Keywords:** Cellobiohydrolase, 1,4-ß-glucanase, Halotolerant, Halophilic, GH6, Salt tolerant, Reversible denaturation, Thermostability, CBH, Sea water, CAZyme, Industrial microbiology, Biocatalysis, Molecular biology

## Abstract

Realising a fully circular bioeconomy requires the valorisation of lignocellulosic biomass. Cellulose is the most attractive component of lignocellulose but depolymerisation is inefficient, expensive and resource intensive requiring substantial volumes of potable water. Seawater is an attractive prospective replacement, however seawater tolerant enzymes are required for the development of seawater-based biorefineries. Here, we report a halophilic cellobiohydrolase SMECel6A, identified and isolated from a salt marsh meta-exo-proteome dataset with high sequence divergence to previously characterised cellobiohydrolases. SMECel6A contains a glycoside hydrolase family 6 (GH6) domain and a carbohydrate binding module family 2 (CBM2) domain. Characterisation of recombinant SMECel6A revealed SMECel6A to be active upon crystalline and amorphous cellulose. Mono- and oligosaccharide product profiles revealed cellobiose as the major hydrolysis product confirming SMECel6A as a cellobiohydrolase. We show SMECel6A to be halophilic with optimal activity achieved in 0.5X seawater displaying 80.6 ± 6.93% activity in 1 × seawater. Structural predictions revealed similarity to a characterised halophilic cellobiohydrolase despite sharing only 57% sequence identity. Sequential thermocycling revealed SMECel6A had the ability to partially reversibly denature exclusively in seawater retaining significant activity. Our study confirms that salt marsh ecosystems harbour enzymes with attractive traits with biotechnological potential for implementation in ionic solution based bioprocessing systems.

## Introduction

Attaining a fully circular bioeconomy requires the efficient and effective utilisation of all biologically derived by-products. Lignocellulosic residues offer the greatest opportunity for a renewable and sustainable feedstock to support the bioeconomy through the production of sustainable fuel, valuable chemicals and sustainable materials. Lignocellulose is a composite of complex polysaccharides in the form of cellulose, hemicellulose and pectins and an aromatic polymer lignin that interconnect to form a complex network of polymers in the secondary plant cell wall. The molecular and structural complexity of lignocellulose confers extreme recalcitrance requiring extreme pre-treatments for efficient enzymatic hydrolysis, however; these processes require a substantial volume of potable water potentially threatening water security local to bioprocessing developments with estimates ranging between 1.9 and 9.8 L of potable water consumed during processing only per litre of ethanol produced, at scale these volumes can have considerable local and regional impacts^1–3^.

A prospective alternative is the use of seawater, an inexhaustible and inexpensive solvent that would liberate expensive and increasingly scarce potable water for agricultural use and human consumption. In doing so, process economics and sustainability would improve as well as mitigating water security issues. Incorporating untreated seawater as the main solvent in bioprocessing systems requires full process level compatibility. Seawater is a complex solvent with approximate pH 8.15 containing around 3.5% (w/v) salt of which 2% comprises of over 82 trace elements existing as diverse chemical species^4–6^ while the two most abundant components are sodium chloride and potassium chloride at 14% and 84% respectively.

Often seawater is an unfavourable solvent for enzymes as in the presence of ionic solutions, many enzymes aggregate, precipitate and lose activity. The mechanisms underpinning halotolerant characteristics are not fully known; however, adaptations include exhibition of increased numbers of acidic surface residues (aspartic acid and glutamic acid) and reduced numbers of lysine residues and reduced abundance of hydrophobic residues (glycine, alanine, valine) that in combination produce a greater negative electrostatic surface potential are a common feature^7–9^. These adaptations putatively facilitate the formation of salt-bridges between the dissolved cationic salt and anionic carboxylate that configures an ordered hydration shell facilitating the dissolution and stabilisation of proteins in concentrated ionic solutions^10–12^. The Hofmeister series describes electro-selectivity of ion and ion-protein interactions causing proteins to salt in and salt out, with ion shielding preventing protein–protein interaction increasing solubility and protein dehydration increasing protein–protein interactions decreasing solubility at low and high ion concentrations respectively. As such, initial resistance to dehydration is a major factor determining tolerance to ionic solutions. In the context of seawater Ca^2+^, Mg^2+^, Na^+^, K^+^, Br^−^ and Cl^−^ are highly abundant destabilisers^13^. More recently, halotolerance is of particular interest due to the emergence of efficient ionic liquid based lignocellulose pre-treatments^14^ with which consolidated bioprocessing using halotolerant cocktails and raw ionic liquid treated biomass has significant valorisation potential.

Cellulose is often the most abundant constituent of lignocellulose that exists as a linear polysaccharide comprised of repeating β-(1–4) *D*-glucose units with successive residues rotated by 180° around the β-(1–4) glycosidic linkages, conferring the disaccharide cellobiose as the basic repeating unit. Crystalline cellulose is formed by tightly packed cellulose chains to form micro fibrils and this crystallinity confers stability in the cell wall and recalcitrance to degradation through inaccessibility and compaction. Cellulases are glycosyl hydrolase (GH) family enzymes that synergistically act to depolymerise cellulose but differ substantially in their modes of action, interaction with cellulose and products produced. Cellulases are present in families GH1, GH3, GH5-9, GH12, GH44-45, GH48 and GH74^15^. Endoglucanases (EC 3.2.1.4) perform cleavage events at internal β-1,4-glucan positions within the cellulose chain producing free ends, multiplying assessable initiation sites for exo-acting cellulases. Exoglucanases (EC 3.2.1.91 and 3.2.1.176) are collectively known as cellobiohydrolases (CBH) at the reducing ends (CBH-I) or non-reducing ends (CBH-II) that act progressively to perform cleavage events at the repeating units producing cellobiose and β-glucosidases (EC 3.2.1.21) hydrolyse cellobiose into glucose. Lytic polysaccharide monooxygenases (LPMOs; EC 1.14.99.54 and EC 1.14.99.56) currently exist as auxiliary activity (AA) families AA9, AA10, AA15 and AA16 are metalloenzymes that require electron donors to orchestrate oxidative glycosidic cleavage events^16,17^.

The seawater based biorefining concept has additional benefits greater than reducing water requirements including cost savings, improved land use efficiency, flexibility in facility locations and mitigation of environmental impacts^18^. Seawater also has processing advantages, passive seawater treatments on lignocellulosic rice straw and rice husk have shown a significant reduction in hemicellulose (19% and 12.4% respectively) and lignin (6.1% and 9.3% respectively) with physical changes in roughness, flexibility and crystallinity^19^. While more intensive hydrothermal lignocellulose pre-treatments have shown effective removal and degradation of hemicellulose putatively catalysed by the diversity of salts present^20–22^, concordant with high sodium chloride treatments^23–25^ and seawater infused ionic liquid pre-treatments^26^. Utilisation of these findings could alleviate industrial scale harsh physio-chemical pre-treatments and their associated adverse environmental impacts. Early investigations demonstrating the seawater based biorefining concept have ranged from fermentations targeting ethanol production from *Kluyveromyces marxianus* and artichoke in seawater mixtures^27^ to more conventional approaches using seawater-based sugarcane molasses fermentations^28^. The concept has been assessed in the context of more complex lignocellulosic feedstocks such as rice straw, palm residues, sugarcane bagasse, papaya and the use of macroalgae and microalgae^13,21,29–34^. However, significant further comprehensive research is required to advance the seawater-based biorefinery concept. One area of focus is to identify an expanded repertoire of halophilic enzymes as the majority of biorefining research has focussed on non-ionic based solvents using enzymes from terrestrial systems, therefore it is likely optimal saccharification in seawater has yet to be achieved.

Despite cellulases being ubiquitous across the tree of life, natural diversity for these enzymes and particularly for their halotolerant representatives have yet to be fully explored. This is due in part to the scarce number of examples where the saline environments and lignocellulose derived polymers co-exist where selection pressures have evolved adaptions to ionic solutions in lignocellulolytic enzymes. In addition, halotolerant adaptations confer deep sequence level divergence between non-salt tolerant enzymes and salt tolerant enzymes that restricts identification. For this reason, annotation reliability for halotolerant enzymes using archive databases is often inadequate due to low levels of representation particularly for high confidence annotations from characterisation-level proteins. The GH6 family CBH in particular is poorly represented in terms of functionally characterised proteins^35–37^ with even fewer reports of halotolerant variants^38,39^. A correlation between thermostability and halotolerance has long been known eluded to^40–42^ and recent engineering approaches have yielded simultaneous improvements in CBH thermostability and tolerance to ionic liquids and seawater^42^. These findings strengthen the need for a greater depth of representative sequences for use as templates in engineering studies and further discovery, an approach that holds promise given the success of previous cellulase engineering efforts^43^.

In this study, we report a novel exoglucanase with significant sequence level divergence to characterised proteins but with significant structural similarity, SMECel6A; that contains GH6 and CBM2 domains identified using a targeted meta-exo-proteomic approach. We describe the cloning, recombinant expression, purification and biochemical characterisation of SMECel6A. We demonstrate SMECel6A as a cellobiohydrolase (CBH) which is halophilic with optimal activity 0.5X seawater, consistent with the environmental origin, and tolerant to high salt conditions up to 3M NaCl. SMECel6A displays considerable plasticity in its active pH (4–7.5) and temperature range (25–50 °C). We also demonstrate considerable thermostability with reversible denaturation with retained activity of SMECel6A exclusively in the presence of seawater not observed in ion-free solution.

## Materials and methods

### Heterologous expression, purification and characterization

An open reading frame (a_c570829_g1_i1_1) containing glycoside hydrolase family 6 (GH6) and carbohydrate binding module family 2 (CBM2) annotation, referred to here as SMECel6A, was identified as the most abundant CAZyme within a salt marsh exo-meta-proteome dataset^44^. A signal peptide was identified from the open reading frame SMECel6A using SingalP^45^ which was trimmed from the coding sequence between positions 28 and 29: AQA-AS and amplified from genomic DNA harvested from Welwick salt marsh (Hull, UK) using forward GCATCTTGTTCGTATTCATTGA (5′–3′) and reverse TTCCGCAGGAGGACCTG (5′–3′) primers. The amplicon was cloned into pET-52b( +) expression vector with an introduced N-terminal streptavidin II affinity peptide tag (WSHPQFEK) and transformed into *Escherichia coli* Rosetta-gami™ 2 (DE3) competent cells (Novagen). The full sequence is available under GenBank accession OR614076. Bacterial cultures were grown at 175 rpm at 37 °C and heterologous expression was induced at A_600nm_ of 0.6 with 0.1mM isopropyl ß-D-1-thiogalactopyanoside (IPTG) at 16 °C for 16 h. Protein purification was performed using a StrepTrap™ HP column (GE healthcare) with fast protein liquid chromatography system (FPLC, GE Healthcare) and protein was eluted with 2.5mM d-desthiobiotin at 1 mL min^−1^.

### Reducing sugar assay

Reducing sugars were detected using 4-hydroxybenzoic acid hydrazide (PAHBAH)^46^. Sugar hydrolysate was incubated with a solution containing 50mM PAHBAH, 10mM bismuth nitrate, 10 mM sodium, potassium tartrate in 0.5M NaOH at 72 °C for 10 min. Absorbance changes were detected at 405 nm and quantified using glucose standards and against relevant boiled enzyme or no enzyme controls.

### Substrate affinity and activity

A range of complex polysaccharide, oligosaccharide and respective derivatives were used to determine substrate specificity. Reactions were performed for 6 h at 250 rpm with 39.4nM SMECel6A (3µg mL^−1^) at 43.8 °C and 50mM sodium acetate pH 5.2. The polysaccharides utilized and their final concentration (w/v) in reaction mixtures included; 0.5% Avicel™ (m/v; Sigma), 1% (m/v) phosphoric acid swollen cellulose (PASC), 0.5% carboxy-methyl cellulose sodium salt (m/v; Sigma), 0.5% (m/v; Megazyme) beechwood xylan (1–4-ß-xylose), 0.1% (m/v; Megazyme) barley glucan (1–4-ß-glucan), 0.1% (m/v; Megazyme) lichenan (1,3:1,4-ß-glucan, 0.1% (m/v; Megazyme) pachyman (1,3-ß-glucan), 0.1% (m/v; Megazyme) arabinoxylan, 0.1% (m/v; Megazyme) arabinogalactan, 0.1% (m/v; Megazyme) xyloglucan, 0.1% (m/v; Megazyme) Lupin galactan, 0.1% (m/v; Megazyme) glucomannan, 0.1% (m/v; Megazyme) galactomannan, 0.1% (m/v; Megazyme) mannan (1–4-ß-mannan). Activity was determined using the PAHBAH method^46^.

Mono- and oligosaccharides (glucose, cellobiose, cellotriose, cellotetraose, cellopentaose and cellohexaose; Megazyme) were utilized at a final concentration of 0.1mM or 0.5mM with 131nM SMECel6A (10 µg mL^−1^) in a final volume of 100 µL for 1 h. Determination of monosaccharide and oligosaccharide product profiles was achieved using ion exchange chromatography using an ICS-3000 Ion Chromatography System (Dionex, Thermo, UK) against relevant standards after^47^. The 4-nitrophenyl-ß-D-R (Carbosynth Limited) fluorometric derivatives (pNP-ß-D-glucopyranoside, pNP-ß-D-cellobioside, pNP-ß-D-cellotrioside, pNP-ß-D-cellotetraoside, pNP-ß-D-cellotetraoside and pNP-ß-D-cellopentaoside) were utilized in a final concentration of 0.5mM as above. Reactions were terminated with the addition of 1M sodium carbonate (Na_2_CO_3_) to a final concentration of 0.5M. Activity was assessed against 4-nitrophenol standards and measured at 405nm.

### Bivariate pH, temperature optima

Two-dimensional pH and temperature experiments were conducted in triplicate with relative activity profiles obtained using a 100 mM citrate–phosphate (CPB) buffer system in a pH range of 4–8^48^. The reaction mixture contained 13 1nM SMECel6A (10 µg mL^−1^) and 2% PASC for 24 h in a total reaction volume of 100 µL. Reducing sugar release was quantified against a boiled enzyme negative control with the PAHBAH method.

### Halotolerance

Salt tolerance was determined using artificial seawater (ASW; with typical ionic concentrations at 1 × concentrate: Chloride 19,336 (mg/L), Sodium 10,752 (mg/L), Sulfate 2657 (mg/L), Magnesium 1317 (mg/L), Potassium 421 (mg/L), Calcium 380 (mg/L), Carbonate/Bicarbonate 142 (mg/L), Strontium 9.5 (mg/L), Boron 16 (mg/L), Bromide 56 (mg/L), Iodide 0.060 (mg/L), Lithium 0.3 (mg/L), Silicon < 0.1 (mg/L), Iron 0.0098 (mg/L), Copper 0.0003 (mg/L), Nickel < 0.015 (mg/L), Zinc 0.0107 (mg/L), Manganese 0.0023 (mg/L), Molybdenum 0.0098 (mg/L), Cobalt 0.0004 (mg/L), Vanadium < 0.015 (mg/L), Selenium < 0.019 (mg/L), Rubidium 0.118 (mg/L), Barium 0.04 (mg/L) (Marine salt, SeaChem, UK) and sodium chloride concentrations equivalent to the ionic strength of estuarine waters, seawater and above. Enzyme preparations were dialyzed three times in 5 000 × volume of > 18 MΩ·cm dH2O for 2, 16 and 2 h respectively to remove ionic background. Reactions were conducted at the respective pH and temperature optima; 43.8 °C and 50mM sodium acetate pH 5.2 in 0.5% Avicel™ with 131nM SMECel6A (10 µg mL^−1^) for 6 h at 250 rpm.

### Thermostability

Thermal shift assays were performed using SYPRO™ orange protein dye (Sigma S5692) at a final dye concentration of 1–5 × and assayed on a Stratagene Mx300 rtPCR for 70 cycles between 25 °C and 95 °C at increments of 2 °C min^−1^. Fluorescence data was normalised to elucidate the protein melting point (Tm). Thermostability assays were conducted by heat treatment of SMECel6A at 100 °C in various ionic solutions followed by 1h of renaturation at 25 °C. Sequential thermostability and reversible renaturation of SMECel6A was explored in a Prometheus nanoDSF (Nanotemper Technologies, London, UK) at 13.1uM (1mg/mL) in NT.48 standard capillaries during ten thermocycles between 25 °C for 25 min and 90 °C for 5 min at a ramp rate of 1 °C min^−1^ at pH 5.2 in sodium chloride, artificial seawater and 100 mM citrate phosphate buffer. Denaturation and renaturation were assessed as an index of intrinsic tryptophan fluorescence at 350nm/330nm. Precipitation was assessed as a function of light scattering (mAU). Confirmation of function post heat treatment was validated using the PAHBAH method using 0.5% Avicel™ with 126nM SMECel6A (10 µg mL^−1^).

### Processivity

Avicel (0.2%), phosphoric acid swollen cellulose (0.2%) and carboxy-methyl cellulose (0.05%) at 13.12nM SMECel6A (1 µg mL^−1^) for 1 h at optimum conditions. To sample, the reaction was centrifuged at 4600 rpm for 1 min and 4 volumes of 100% 4 °C ethanol added to precipitate proteins and impurities. The hydrolysate was dehydrated in a refrigerated vapor trap (Thermo). Samples were re-suspended in the original volume taken and analysed with ion exchange as above. Apparent processivity (P_app_) was determined semiquantitatively as the molar ratio of cellobiose/(glucose + cellotriose)^49,50^.

### Structural analysis and predictions

The structure of CMECel6A was predicted using AlphaFold version 2.1.1^51^. Five models were generated with a mean pLDDT of 91.72 ± 0.75; the model selected for analysis exhibited a pLDDT of 92.54. Structural alignments and electrostatic potentials were generated using ChimeraX^52^.

### Bioinformatics and statistics

Analysis of Variance (ANOVA) and Kruskal–Wallis H tests were performed using SciPy^53^ and Scikit^54^. All data was assessed for normality using the Shapiro–Wilk test.

## Results

### Protein production and properties

A multi-domain protein originating from open reading frame a_c570829_g1_i1_1^44^, designated here as SMECel6A, was identified as the most abundant CAZyme within a salt marsh meta-exo-proteome dataset^44^. SMECel6A has maximum identity of 50% to a characterised *Cellulomonas fimi* 1,4-ß-cellobiohydrolase (Swiss-Prot accession P50401.1). SMECel6A is multi domain protein containing a carbohydrate-binding module (CBM) family 2 domain (IPR012291) and glycoside hydrolase (GH) family 6 domain (IPR016288) separated by two sequential predicted PKD domains (IPR035986) with three predicted disulphide bridges ostensibly acting as a linker between the CBM2 and GH6 domains. Recombinant expression was performed using a pET-52b( +) expression vector in *Escherichia coli* Rosetta-gami™ 2 (DE3) cells and purified to homogeneity using an introduced N-terminal streptavidin affinity tag. The recombinant protein exhibits a predicted molecular weight of 76.1kDa and theoretical isoelectric point (pI) of 4.32.

### Substrate specificity, optima, and product profiles of SMECel6A

**Figure 1:**
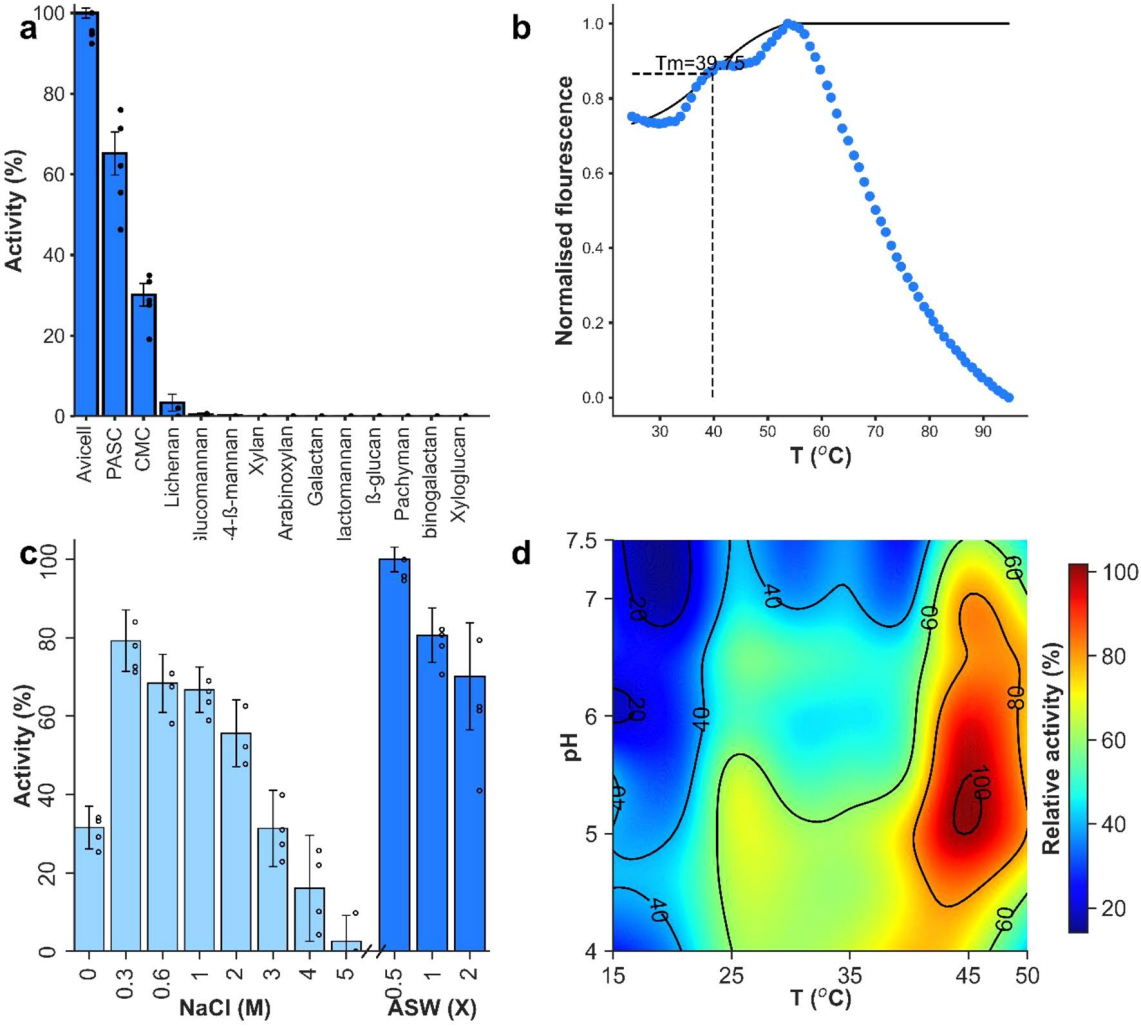
Characteristics of SMECel6A. (**a**) Substrate affinities of SMECel6A. Error bars represent standard error of five technical replicates. Asterisks indicate significance at a confidence interval of *p* =  < 0.05 assessed by a one-way ANOVA. (**b**) A representative thermal shift profile of SMECel6A. A sigmoidal curve between the maximum and minimum before the maximum is shown, the median value of this fit was determined as the melting point (Tm; 39.75). (**c**) Halotolerance of SMECel6A as a function of activity in solutions of increasing salt concentrations. (**d**) Bivariate pH/temperature optima of SMECel6A (optima; 43.8 °C, pH 5).

The hydrolytic activity of SMECel6A was tested against 4-nitrophenyl-ß-D-R glucooligosaccharide derivatives (pNP-ß-D-glucopyranoside, pNP-ß-D-cellobioside, pNP-ß-D-cellotrioside, pNP-ß-D-cellotetraoside and pNP-ß-D-cellopentaoside) but no detectable activity was observed after incubation for 24h. However; screening of polysaccharides revealed substantial sugar release and a high affinity for Avicel™ that was significantly higher than both PASC (ANOVA, *F*_1,8_ = 36.7, *p* < 0.00) and CMC (ANOVA, *F*_1,8_ = 479.35, *p* < 1.99 × 10^−8^) with relative activities of 62.25 ± 10.7% and 28.75 ± 5.57% respectively. Insignificant activity (Kruskal–Wallis test: H = 1, DF = 1, *p* = 0.317) was also detected for lichenan (1,3:1,4-β-D-Glucan) and glucomannan (Fig. 1a).

To assess the optimal pH and temperature conditions for SMECel6A, a bivariate experiment was undertaken which elucidated the optima as 43.8°C and pH 5.2 (Fig. 1d). Interestingly, both the pH and temperature had a broad range of high functioning in which the enzyme remained highly active between pH 4 and 7.5 and retained 60% activity between 50 °C and 25 °C. A thermal shift assay was performed to confirm the structural integrity of GH6 and CBM2 domains, revealing two unfolding events (Fig. 1b) and these observations are also confirmed by the bivariate pH and temperature optima assessment profile which displayed two activity apices in agreement with the double peak observed within the thermal shift profile suggesting two independent unfolding events with independent effects on catalytic efficiency (Fig. 1b). The melting point (Tm) was calculated to be 39.75 °C however this is likely biased lower due to the double peak.

**Figure 2:**
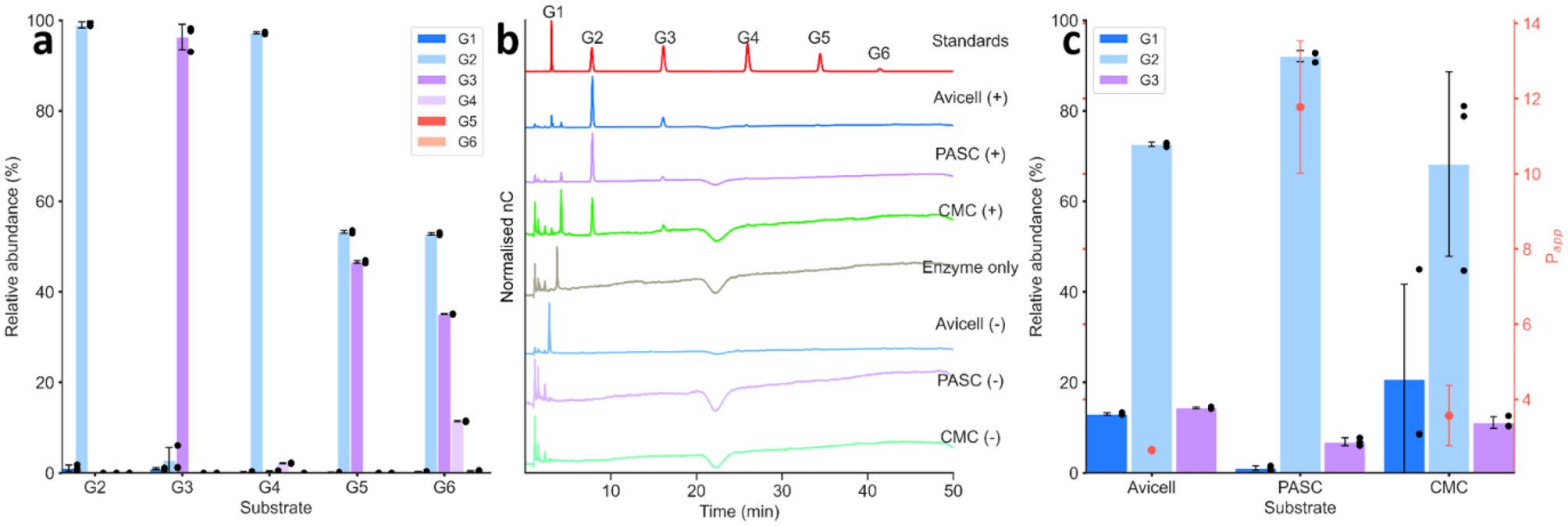
Product profiles for SMECel6A post reaction with oligosaccharides substrates. (**a**) Product profile SMECel6A after incubation with oligosaccharides. (**b**) Ion exchange chromatography product profiles SMECel6A after incubation with polysaccharides. (**c**) Relative abundance of products and apparent processivity (P_app_) after incubation with Phosphoric acid swollen (PASC), Carboxy-methyl cellulose (CMC) and Avicell™. G1; glucose, G2; cellobiose, G3; cellotriose, G4; cellotetraose, G5; cellopentaose, G6; cellohexaose.

To determine the mode of action for SMECel6A, product profiles were elucidated using ion exchange chromatography after incubation with monosaccharides and oligosaccharides (G1–G6). It was apparent SMECel6A was unable to hydrolyse G2 and showed very weak activity toward G3 with negligible quantities of G1 and G2 detected during incubation (Fig. 2a). The major product from incubation with G4 was cellobiose, incubation with G5 evolved only G2 and G3, and incubation with G6 revealed only G2, G3 and G4. Further product profiles were determined for polysaccharides where activity was highest (Avicel™, PASC and CMC; Fig. 2b). In all cases, synonymous with results observed with oligosaccharide substrate, the major product G2, with G1 and G3 evolved as minor products in all reactions. The product profiles for each polysaccharide was quantified and relative proportions were used to determine enzyme apparent processivity (P_app_) on each substrate which were determined to be 2.65 ± 0.06, 11.77 ± 1.76 and 3.57 ± 0.80 for Avicell™, PASC and CMC respectively (Fig. 2c).

### Salt tolerance, thermal stability and reversible renaturation

Using the optima determined above, SMECel6A tolerance to ionic conditions was explored using NaCl and artificial seawater (ASW). Activity was significantly higher in ionic reaction media compared to dialyzed H_2_O conditions where 31.6 ± 5.45% relative activity was observed (Fig. 1c). Activity was highest in 0.5X seawater and significantly higher than the highest activity observed in NaCl (0.3M) where 79.2 ± 7.8% activity was retained. Activity remained high in high ionic strength solutions up to 2M NaCl (55.6 ± 8.45%) before declining rapidly in 3M (31.34 ± 9.76%) and 4M (16.05 ± 13.54%) NaCl solutions, however higher artificial seawater concentrations retained higher levels of activity at and for 1X (80.86 ± 6.93%) and 2X (70.17 ± 13.62%) concentrated seawater respectively.

**Figure 3:**
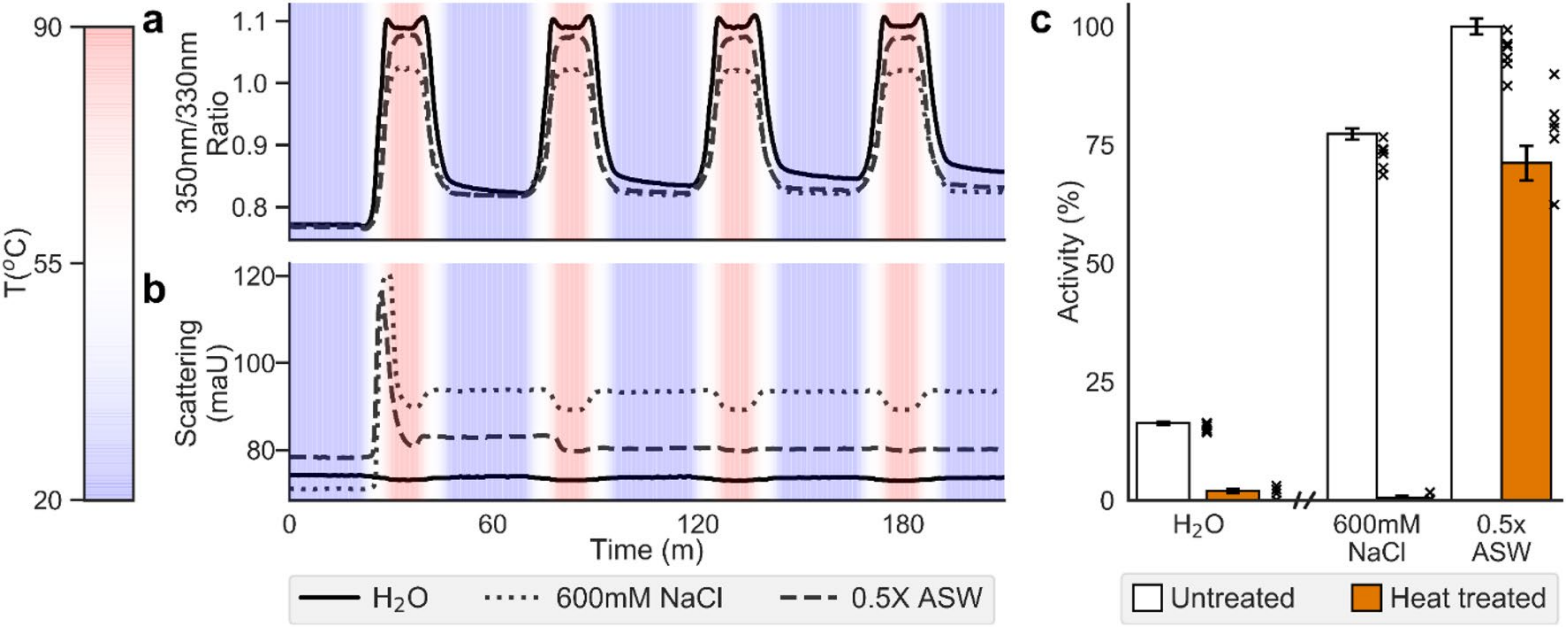
Thermal reversible denaturation profile of SMECel6A. (**a**) Thermal shift profile as a function of fluorescence at 330nm and 350nm. The enzyme was subjected to four sequential 20 °C to 90 °C treatment cycles. The fluorescence ratio increases as the protein denatures. The results show that the thermal shift profile was notably different between H_2_O and ionic solutions. (**b**) Light scattering as a function of enzyme aggregation for enzyme SMECel6A when subjected to four sequential 20 °C to 90 °C treatment cycles. Scattering increases as proteins precipitate. Partial and almost complete re-solubilisation is visible for 600mM NaCl and 0.5X ASW solutions respectively. (**c**) Relative activity of untreated and heat treated SMECel6A, means are displayed as re-normalised percentages, raw data is plotted individually. *ASW*: artificial seawater. Error bars represent SE (n = 6).

The observation that salt is required for optimal performance was explored further during thermostability assessments. We observed a resistance to inactivation post heat treatment of SMECel6A in complex ionic solutions. Consecutive thermal cycling between 5 and 90 °C revealed a profile suggestive of reversible denaturation. The thermal shift profile was notably different between ultrapure H_2_O and ionic solutions (Fig. 3a,b). Irreversible protein aggregation as measured as light scattering (maU) during thermal cycling was observed in sodium chloride solutions; however, in seawater the protein appeared to largely return to solution upon cooling. No aggregation was observed in the ultrapure H_2_O condition, however, activity assays performed with the heat- treated enzyme revealed almost complete inactivation for H_2_O and NaCl conditions, while the enzyme retained 71.2 ± 3.67% 0.5X concentrated seawater (Fig. 3c).

**Figure 4:**
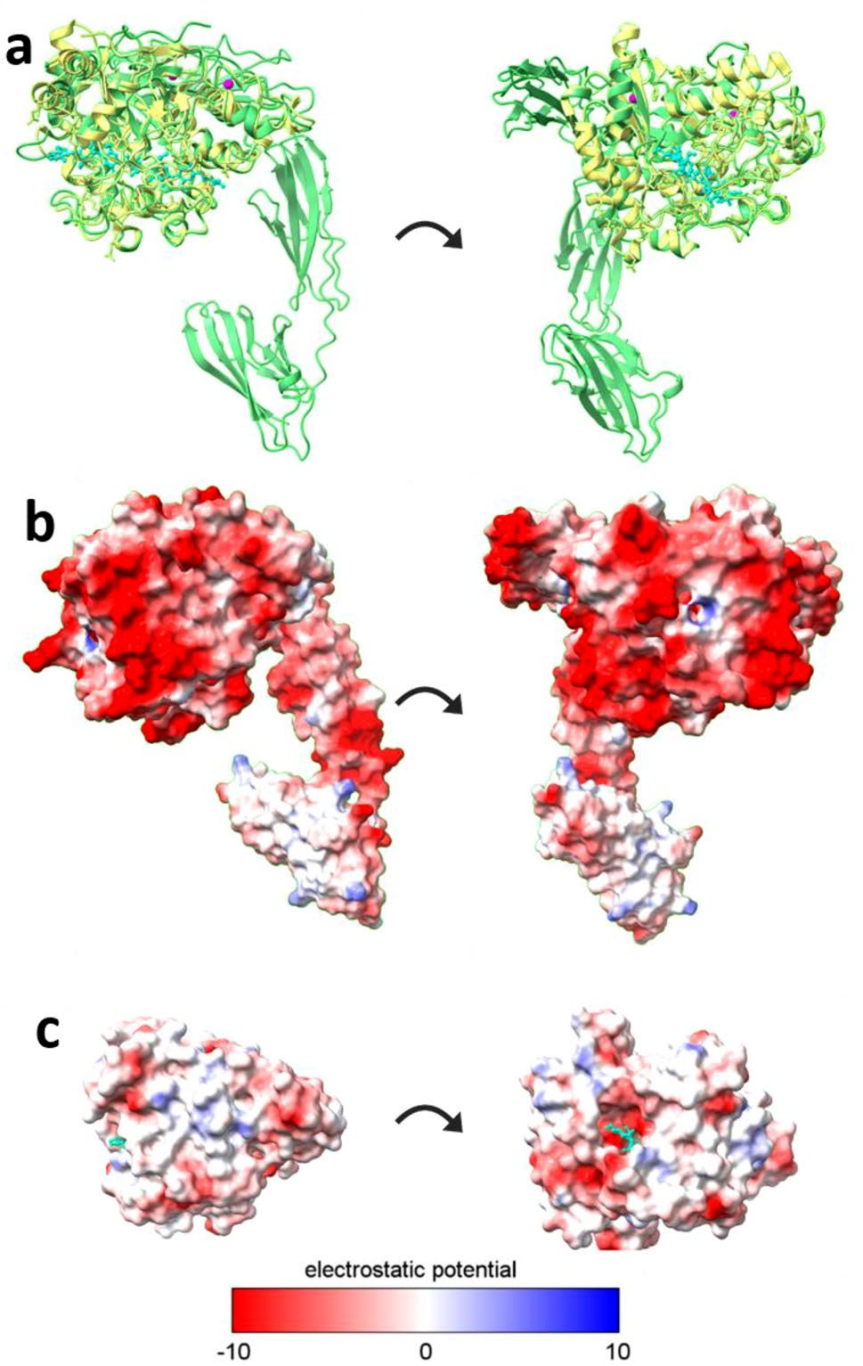
Predicted structure and structural comparisons of SMECel6A. (**a**) Structural alignment between SMECel6A (cellobiohydrolase domain and carbohydrate binding module family 2; green) and HmCel6A (10.2210/pdb6K54/pdb; yellow) in complex with trisaccharide (cyan). Calcium ions are displayed in pink. (**b**) Electrostatic potential of SMECel6A shown as a colored gradient between red (acidic) to blue (basic). (**c**) Electrostatic potential of HmCel6A shown as a colored gradient between red (acidic) to blue (basic).

Despite major sequence level divergence, there were significant structural similarities between HmCel6A and the predicted structure of SMECel6A (Fig. 4a) with calculated root mean square deviation (RMSD) value of 0.655Å for the CBH domains. Analysis of surface residue properties reveals a marked predominance of negatively charged residues as expected from highly ion-tolerant protein (Fig. 4b). Surface charge analysis suggests that SMECel6A exhibits a highly negatively charged surface greater than that of its most similar counterpart HmCel6A (Fig. 4b,c). Both of which exhibited a disproportionate abundance of negative and neutral charges coupled with a distinctive lack of positively charged residues.

## Discussion

The GH6 family comprises exo-1,4-ß-glucanases (cellobiohydrolases; CBH) and endo-1,4-ß-glucanases (endoglucanase; EG). These activities, together with ß–glucosidases and lytic polysaccharide monooxygenases (LPMOs) act to synergistically depolymerise cellulose into smaller oligosaccharides. Here, we explored SMECel6A, a CBM2 containing GH6 family enzyme identified from an environmental meta-omics analysis of a lignocellulosic decomposition performed in situ in a salt marsh ecosystem^44^. Our investigations of SMECel6A revealed product profiles in which G2 was the major product upon hydrolysis of oligosaccharide and polysaccharide substrates suggesting a processive mechanism, while G1 and G3 were identified at trace levels, putatively originating from initial or false start events^55^.

Semiquantitative delineation of apparent processivity (*P*_*app*_), the efficiency and ability with which an enzyme can perform successive successful hydrolytic events prior to dissociation or obstruction^56^; processive catalysis is thought to be advantageous over distributive catalysis for biopolymeic substrates with processive catalysis having been reported in nucleases, proteases and motor proteins as well as exo- and endo- acting glycoside hydrolases, despite this, values are not widely reported ^57^. The *P*_*app*_ for SMECel6A revealed a significantly higher processivity for the hydrolysis of amorphous cellulose (PASC; 11.77 ± 1.76) than crystalline (Avicell™; 2.65 ± 0.06) or soluble cellulose (CMC; 3.57 ± 0.80). The observed disparity likely arises from the greater accessibility of amorphous cellulose microfibrils as processive enzymes complex with the chain terminus^58^ reducing the number of false start events. These results fall within the spectrum of previously determined values for CBH belonging to broad families of 2.7–20.8^49,59–63^. The P_app_ of SMECel6A for crystalline cellulose are lower than previously reported bacterial CBH, such as wild type TfCel6B (*Thermobifida fusca*) with P_app_ values of 7.2–11.8 on crystalline cellulose (filter paper)^59,62^, as well as LqCel7b (*Limnoria quadripunctata*) and Cel7D (*Phanerochaete chrysosporium*) where a P_app_ values greater than 7 were recorded on PASC and Avicel^50,64^.

The affinity of SMECel6A for crystalline cellulose may be due to the CBM2 domain which is known to bind crystalline cellulose and are thought to enhance catalytic activity by retaining proximity and interface with the substrate which could explain the dual peak in activity profiles during the assessment of pH and temperature optima^65^. This feature may be particularly important for this enzyme originating from a highly turbid salt marsh environment to prevent the loss of enzyme from the originating location to retain the benefit to the secreting organism.

These results are concordant with previous observations, where GH6 family cellobiohydrolase catalytic domains are known to be highly processive on crystalline cellulose such as CfCel6B^66^. However, the relationship between processivity and efficiency with regard to turnover and rates of reaction are complex and not yet fully understood^67^, evidenced here by the significantly higher rates of activity on crystalline cellulose where processivity was observed to be lowest (Fig. 2c). Our results suggest SMECel6A is a processive CBH and CBHs within the GH6 family putatively act from the non-reducing ends of cellulose (EC 3.2.1.91)^68^. Similar substrate affinities reported here have been observed for other GH6 family cellobiohydrolase, such as PcCel6A from *Paenibacillus curdlanolyticus* B-6^36^ but also contrasted by CBM3 containing cellobiohydrolase Cel6D from *Paenibacillus barcinonensis* that displayed almost exclusive affinity for amorphous cellulose ^37^.

Cellobiohydrolases occupy 40–60% of lignocellulolytic cocktails^69^ which signifies their importance, despite this there are few known salt tolerant cellobiohydrolase representatives^50,70–72^, ostensibly due to a lack of assessment and limited interaction of cellulose in extreme saline environments restricting the pool of candidates for investigation as few saline environments, with the exceptions of salt marshes, seagrass meadows and mangroves, contain reliable supplies of pristine lignocellulose. Salt marshes vary greatly in their form, biotope and salinity and exist in sheltered intertidal regions transitioning the terrestrial to marine environment that are characterised by halotolerant fauna and flora with high primary productivity. Their periodic inundation and disturbance with seawater mediates an array of physicochemical stresses that vary in extremity on diurnal, seasonal, and inter-annual timescales in relation to other factors (air exposure, precipitation levels, seawater nutrient loading, tidal cycle), which underpin huge surface level fluctuations in pH (below 7.2–8.3 and above), salinity (2–3% and above), temperature (sub-zero to 25 °C and greater), pCO_2_ and O_2_ (1–14 mg L^−1^) with heterogeneous pockets of greater extremities^73^. It is therefore probable that enzymes from these ecosystems have tolerances reflective of their native environment that confers these ecosystems as valuable resources for bioprospecting. Instead, structure-based engineering approaches have been employed to enhance catalytic activity, thermal and pH stability and ionic tolerance with various degrees of success^42,74^. Similar plasticity in environmental tolerance been observed for an endo-acting halophilic GH6 isolated from *Thermobifida haloterans* YIM 90,462(T)^75^. Our observations of SMECel6A, where optimal activity can only be achieved in ionic solutions designate the enzyme as halophilic. Halophilic enzymes commonly feature adaptations that reduce hydrophobicity, increased proportions of acidic surface residues particularly aspartic acid, reduced cysteine residues and tendencies to form coiled structures over helixes^76,77^. Often, these adaptations confer polyextremophilic properties where resistance to one environmental extreme also confers resistance to others, mediated by the presence of salt ions^78^. In support of this, for SMECel6A these adaptations also appear to have consigned great plasticity in pH and temperature tolerances within which high activity is retained across a broad range.

Exo-proteomic approaches are advantageous as they are culture independent and enable the identification of proteins in environmental conditions in situ. Indeed, similar approaches have yielded successful results, the crystal structure of a novel hyper-thermostable cellobiohydrolase, HmCel6A (10.2210/pdb6K54/pdb) was the highest identity match to SMECel6A GH6 domain at 57% was identified by employing a metagenomics mining approach from extreme environments^39^. This exemplifies the degree to which sequence divergence exists for enzymes among and between extreme environments and strengthens the case for further exploration. Despite these difference, structural homology between HmCel6A and SMECel6A was strikingly high suggesting a high degree of compositional heterogeneity within the natural diversity within and among halophilic proteins.

Taxonomic delineation of SMECel6A, originating from a meta-exo-proteomic dataset is challenging as the parent organism cannot be precisely determined, however, SMECel6A has significant nucleotide identify (> 80%; CP033077.1) to *Vibrio spp.,* also confirmed within the original dataset^44^. The protein also contains a CBM2 domain, prevalence of which outside of bacteria is rare, supports this hypothesis^79^.

The assessment of biocatalysts and in particular cellulases in seawater as a hydrolytic solvent to date is limited. Seawater concentration has been shown to be generally inhibitive to cellulase activity, with the enzyme-dependent inhibitory effects. Wild type CelA2 lost > 50% activity in seawater, where directed evolution generated a mutant with a 1.6-fold relative improvement in activity^80^. Industrial cellulase cocktails, including Accellerase-1500 have been shown to be effective on amorphous and crystalline cellulose but exhibited minor inhibitory effects^81^. In accordance with this, the production of seawater optimised cocktails has been explored using halophilic organisms in seawater-based production media such as *Fusarium subglutinans* MTCC11891 that produced thermostable, pH stable, and halophilic cellulases with optimal activity observed 0–8-1M NaCl^82^. Halophilic organisms such as *Aspergillus spp.* have also shown promising halophilic enzyme products such as cellulases from *Aspergillus flavus* tolerant up to 200 g L^−1^ NaCl^83^, endo-1,4-β-xylanase from *Aspergillus* clavatus tolerant to 3M NaCl^84^ and cellulases and xylanases from halophiles *Aspergillus flavus* and *Aspergillus penicilloides* co-culture tolerant up to 30% NaCl^85^.

Exposing SMECel6A to sequential denaturing thermal treatments followed by a period of rest to facilitate renaturation revealed reversible denaturation ability for CMECel6a exclusively in seawater conditions. While some precipitation and possible conformational changes were observed, the degree of retained activity after the first thermocycle suggested the observed conformational changes were only partially deleterious and likely associated with the CBM2 domain that was poorly thermostable in pH and temperature optimisation experiments (Fig. 1d). It is possible the reduced rate of activity is a result of the loss of the CBM2 domain, observed in the pH and temperature optima profile to boost activity, reducing the overall rate of activity and that the catalytic domain was largely unaffected. Additionally, refolding of halophilic proteins can take several days and so the 1 h period employed in this study may have been inadequate to facilitate complete refolding^86^. This confirms that ions in seawater play a protective role, supported by our observation that tertiary structure was differentially affected during denaturation in H_2_O, NaCl and ASW conditions (Fig. 3a), putatively from dehydration for the catalytic domain, from dehydration, denaturation and aggregation^41^. The lack of aggregation in ultrapure H_2_O is an interesting observation, it is also possible that exposed hydrophilic regions in the denatured form are sufficient to maintain solubilisation, or that exposed hydrophobic regions become solvated, maintaining solubility. The lack of activity, taken together with the lack of possible ionic interactions that usually promote aggregation and precipitation of proteins, and the large degree of conformational change observed during thermocycling compared to the ionic solutions (Fig. 3a) suggest the formation of a destabilised intermediary complex that changes conformation during thermocycling while retaining solubility in ultrapure H_2_O.

An adaptation of halotolerant enzymes to combat initial dehydration is to display a greater resistance to initial dehydration, likely a result of a greater negative surface charge characterised by acidic surface residues. This feature has been suggested to promote function and maintains solvation in lower water activity environments however the mechanism is not yet fully understood^87^. The negative surface charge improves the ability of halophilic proteins to bind hydrated cations, where they would otherwise disrupt the ordered solvation shell, that help maintain a surface hydration layer, reducing hydrophobicity and preventing denaturation and aggregation^88^. Recent rational design experiments of surface charge engineering of a β-glucosidase have demonstrated these features to have significant effects on stability and catalytic capacity highlighting both the complexity and importance of this characteristic^89^. These features are apparent in SMECel6A which displayed a higher negative surface charge with a greater number of negative and neutral surface residues. This is known to be a shared feature among extracellular enzymes identified from a diversity of taxa including archaea, bacteria and fungi, spanning broad functionalities including lipases, proteases and lignocellulose degrading enzymes with multiple industrial uses^90–92^. The SMECel6A negative surface charges shares similarities to other halophilic cellulases including *Hu*-CBH1 from *Halorhabdus utahensis* that was stable in high concentrations of the ionic solvent 1-allyl-3-methylimidazolium chloride^70^, a halophilic CBH-I from *Penicillium verruculosum* that displayed significant activity in up to 2 times concentrated seawater with minimal inhibition^42^, nmGH45; a halophilic endo-β-1,4-glucanase with negative surface charges demonstrated 88% relative activity in 4M NaCl with similar activity levels in 10% 1-butyl-3-methylimidazolium chloride^93^ and a halophilic and β-glucosidase B9L147 from *Thermomicrobium roseum* that additionally demonstrated significant activity in seawater^94^. Identifying a broader scope of intrinsically halophilic enzymes will also aid future rational engineering efforts.

Here, we determined the mode of action and halotolerance of a divergent bacterial cellobiohydrolase SMECel6A identified using meta-exo-proteomics in a salt marsh ecosystem. SMECel6a has significant industrial potential in the development of consolidated bioprocessing using ionic liquid pre-treatments or seawater based biorefining practices as well as embellishing the available templates from naturally occurring sequence diversity to aid future intelligent design protein engineering studies. The novel sequence, domain architecture and characteristics of SMECel6A will strengthen archive databases to enhance future identification of further halophilic cellobiohydrolases with attractive traits, in particular those with greater negative surface residue charged, which are currently underrepresented using homology and design-based approaches.

## Data Availability

Metaproteomic and metatranscriptomic databases mined during this research are available at MassIVE (https://massive.ucsd.edu/) and ProteomeXchange (http://www.proteomexchange.org/) accessions MSV000083872 and PXD014068 respectively. Sequence data for SMECel6a is available under GenBank accession OR614076.

## Abbreviations

AA: Auxiliary activity enzyme
CAZyme: Carbohydrate-active enzymes
CBH: Cellobiohydrolase
CBM: Carbohydrate-binding module
CMC: Carboxy methyl cellulose
G1: Glucose
G2: Cellobiose
G3: Cellotriose
G4: Cellotetraose
G5: Cellopentaose
G6: Cellohexaose
GH: Glycoside hydrolase
LPMO: Lytic polysaccharide monooxygenase
PASC: Phosphoric acid swollen cellulose
PBS: Phosphate buffered saline
